# Glucosamine-Induced Autophagy through AMPK–mTOR Pathway Attenuates Lipofuscin-Like Autofluorescence in Human Retinal Pigment Epithelial Cells In Vitro

**DOI:** 10.3390/ijms19051416

**Published:** 2018-05-09

**Authors:** Ching-Long Chen, Yi-Hao Chen, Chang-Min Liang, Ming-Cheng Tai, Da-Wen Lu, Jiann-Torng Chen

**Affiliations:** Department of Ophthalmology, Tri-Service General Hospital, National Defense Medical Center, 325 Cheng-Kung Road, Section 2, Taipei 114, Taiwan; doc30881@mail.ndmctsgh.edu.tw (C.-L.C.); keane@ms18.url.com.tw (Y.-H.C.); doc30875@yahoo.com.tw (C.-M.L.); mingtai1966@yahoo.com.tw (M.-C.T.); p310849@ms23.hinet.net (D.-W.L.)

**Keywords:** glucosamine, autophagy, lipofuscin, retinal pigment epithelial cells

## Abstract

Age-related macular degeneration (AMD) is a vision-threatening age-associated disease. The retinal pigment epithelial (RPE) cells phagocytose and digest photoreceptor outer segment (POS). Incomplete digestion of POS leads to lipofuscin accumulation, which contributes to the pathology of the AMD. Autophagy could help reduce the amount of lipofuscin accumulation. In the present study, we evaluated the effects of glucosamine (GlcN), a natural supplement, on the induction of autophagy and POS-derived lipofuscin-like autofluorescence (LLAF) in ARPE-19 cells in vitro, and investigated the potential molecular pathway involved. Our results revealed that GlcN had no effect on phagocytosis of POS at the lower doses. GlcN treatment induced autophagy in cells. GlcN decreased the LLAF in native POS-treated cells, whereas malondialdehyde or 4-hydroxynonenal-modified POS attenuated this effect. 3-Methyladenine inhibited GlcN-induced autophagy and attenuated the effect of GlcN on the decrease of the native POS-derived LLAF. Furthermore, GlcN induced the phosphorylation of AMP-activated protein kinase (AMPK) and inhibited the phosphorylation of mammalian target of rapamycin (mTOR), whereas Compound C inhibited these effects of GlcN. Altogether, these results suggest that GlcN decreased the native POS-derived LLAF through induction of autophagy, at least in part, by the AMPK–mTOR pathway. This mechanism has potential for the preventive treatment of lipofuscin-related retinal degeneration such as AMD.

## 1. Introduction

Age-related macular degeneration (AMD) is a progressive deterioration of the macula of the retina associated with aging. AMD is the chief cause of irreversible vision loss among people aged 50 years and older in the developed world [1]. The prevalence of late AMD, geographic atrophy (GA), and neovascular AMD is 12.2%, 6.7%, and 6.3%, respectively, in those aged 80 years and older [2]. Clinically, AMD is classified into the dry (nonexudative or atrophic) form and the wet (exudative or neovascular) form. The wet form affects approximately 10–15% of all patients with AMD and accounts for 90% of all cases of vision loss associated with the disease [3]. The dry form is more common and affects 85–90% of all patients with AMD. Early stage of dry AMD is associated with mild vision loss and the presence of drusen in the macula [4]. Later, it progresses to GA, causing irreversible vision loss [5]. Therefore, it is important to develop preventive strategies in dry AMD through nutrient supplementation.

Retinal pigment epithelial (RPE) cells form a monolayer of pigmented epithelium, which is located between the neuroretina and the choroid and acts as the outer blood–retinal barrier. They perform many vital functions for supporting the action and survival of photoreceptors, such as phagocytosis and degradation of the photoreceptor outer segment (POS), maintaining the visual cycle, providing multiple nutritional factors, and selectively transporting metabolites between the neuroretina and the choriocapillaris [6]. Consequently, RPE dysfunction can lead to photoreceptor degeneration, which is a crucial molecular event in the pathogenesis of AMD [6].

Lipofuscin is an age-related pigment which accumulates in metabolically active post-mitotic RPE cells and contributes to the pathogenesis of AMD [7]. Unlike other cell types, lipofuscin in RPE cells is derived mainly from the ingestion of incompletely digested extracellular materials and the POS [8,9]. In humans, RPE cells accumulate lipofuscin throughout life and it occupies up to 19% of cytoplasmic volume by the age of 80 years [10]. Even though lipofuscin could be found in both AMD and non-AMD elderly patients, the role of lipofuscin in AMD is still one of the important risk factors. Previously, several studies have identified lipofuscin as a potent photo-inducible generator of reactive oxygen species (ROS) [11,12]. Additionally, it is involved in lipid peroxidation and light-induced enzyme inactivation [13], and is phototoxic to RPE cells through the formation of ROS [14]. Although several studies have suggested that lipofuscin involves certain pathways and is the key in the underlying mechanisms of AMD, it is still unclear how it ultimately causes AMD. Therefore, effective therapeutic strategies to prevent or reduce the accumulation of lipofuscin in RPE cells might be a therapeutic target for AMD.

Autophagy, or “self-eating”, is an important cellular homeostasis process which degrades dysfunctional cellular organelles and protein aggregates through the lysosomal machinery [15]. It plays a significant role in preventing neurodegeneration, age-associated conditions, cancer, and microbial infection [15]. Autophagy is active in the retina and its dysfunction induces transcytosis, exocytosis, and apoptosis in RPE cells [16,17]. In RPE cells, autophagy could be induced by phagocytic uptake of the POS [18]. Autophagy additionally contributes to the clearance of accumulated deposits in RPE cells [19], indicating its importance in the accumulation of lipofuscin in these cells [20]. Thus, autophagy is a potential therapeutic target for decreasing the accumulation of lipofuscin in RPE cells.

Glucosamine (GlcN) is a naturally occurring amino monosaccharide with immunosuppressive effects, both in vitro and in vivo. It is widely used as an alternative therapeutic regimen for rheumatoid arthritis and osteoarthritis [21,22,23]. It is the most common dietary supplement whose long-term administration is considered safe in humans [24]. We previously reported the role of GlcN in the inhibition of inflammatory reactions induced by inflammatory cytokines in human RPE cells; its anti-inflammatory effect in a rat model of endotoxin-induced uveitis [25,26,27]; suppression of epithelial-to-mesenchymal transformation of RPE cells, both in vivo and in vitro [28]; and protection of retinal ganglion cells from oxidative stress injury in a rat model of ischemia–reperfusion injury [29]. GlcN inhibits epidermal growth factor-induced proliferation and cell cycle progression in RPE cells [30]. Recently, researchers have indicated that GlcN activates autophagy in many cell types and has diverse biological properties such as anti-neurodegenerative, anti-aging, anti-apoptosis, and anti-cancer effects [31,32,33,34]. However, the effects of GlcN on autophagy and POS-derived lipofuscin in RPE cells remains unclear. Therefore, the objective of the present study was to investigate the role of GlcN in the induction of autophagy; its influence on the increase of POS-derived lipofuscin-like autofluorescence (LLAF) in RPE cells in vitro; and identify the involved signaling pathways. This work may provide a possible preventive therapeutic strategy against lipofuscin-related retinal degeneration, such as AMD.

## 2. Results

### 2.1. Expression of ZO-1, RPE65, and MerTK Protein in ARPE-19 Cells after One Day and Seven Days of Culture

**Figure 1:**
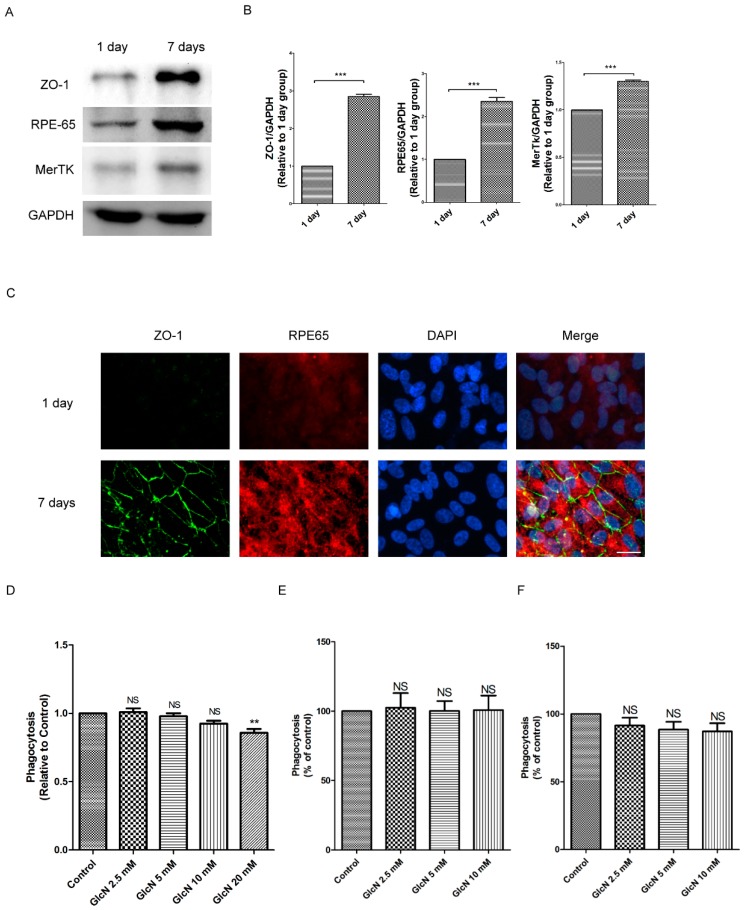
Expression of ZO-1, RPE65, and MerTK protein after one- and seven-day cultures in ARPE-19 cells. (**A**) Western blot analysis detecting the protein expression of ZO-1, RPE65, and MerTK in post-confluent cultures of ARPE-19 cells. The cells were cultured for either one day or seven days. Whole-cell lysates were prepared and analyzed with immunoblotting using anti-ZO-1, anti-RPE65, anti-MerTK, and anti-GAPDH antibodies. (**B**) Quantification of protein expression levels of ZO-1, RPE65, and MerTK. The optical density of the Western blot bands obtained for ZO-1, RPE65, MerTK, and GAPDH were analyzed. The results are represented as the mean ± SEM. The differences in the protein level of ZO-1, RPE65, and MerTK between groups were compared using the paired *t* test. *** *p* < 0.001. (**C**) After one and seven days of culture, the characteristics of RPE cells, including tight junction proteins (ZO-1) and differentiation markers (RPE65) were identified by immunofluorescence staining. Magnification, ×400. Scale bar: 20 μm. Effects of glucosamine (GlcN) on phagocytosis of POS in ARPE-19 cells. (**D**) After seven days of cultures, ARPE-19 cells were pre-treated with or without GlcN (2.5, 5, 10, and 20 mM) for 24 h, followed by co-treatment with fluorescein isothiocyanate-labeled POS (FITC–POS) and the indicated concentration of GlcN (2.5, 5, 10, and 20 mM) for 3 h. The fluorescence intensity was measured using a microplate reader and normalized to the control group. The data are represented as mean ± SEM. ns, not significant; ** *p* < 0.01 versus POS group. (**E**,**F**) After seven days of culture, cells were pre-treated with or without GlcN (2.5, 5, 10, and 20 mM) for 24 h, and then co-treated with FITC–POS and the indicated concentration of GlcN (2.5, 5, and 10 mM) for: 3 h (**E**); and 24 h (**F**). The mean fluorescence intensity was measured by flow cytometry and normalized to control cells. The data are represented as mean ± SEM; ns, not significant versus POS group.

To identify the optimal conditions for the polarized RPE cell culture in our study, we used Western blotting and immunofluorescence staining to study the characteristics of RPE cells. The cells were seeded at 1.66 × 10^5^/cm^2^ and maintained in culture for one day and seven days. Based on a previous study [35], we examined the protein expression of tight junction protein Zonula occludens-1 (ZO-1), visual cycle enzyme RPE65, and phagocytic receptor MerTK in ARPE-19 cells. As shown in Figure 1, Western blot analysis detected significantly enhanced expression of ZO-1, RPE65, and MerTK in seven-day cultures compared to one-day cultures (*p* < 0.001 for each; Figure 1A,B). Consistent with the results of Western blot analysis, immunofluorescence staining revealed significant expression of ZO-1 and RPE65 in seven-day cultures compared to one-day cultures (Figure 1C). Accordingly, seven-day cultured ARPE-19 cells were used for further experiments.

### 2.2. Effect of GlcN on Phagocytosis of POS in ARPE-19 Cells

Previous studies have reported phagocytosis of POS as the major source of lipofuscin in RPE cells [8,9]. To determine the effect of GlcN on POS-derived LLAF, we first investigated the effect of GlcN on the phagocytosis of POS in RPE cells. Fluorescein isothiocyanate-labeled POS (FITC–POS) was used to evaluate the effect of GlcN on phagocytosis in ARPE-19 cells and evaluated by a microplate reader. As shown in Figure 1D, compared to the POS group, there was no significant difference on phagocytosis of POS in co-treatment with indicated concentrations of GlcN (2.5, 5, and 10 mM) and the POS group after being incubated for 3 h. By contrast, compared with the POS group, the phagocytosis of POSs was significantly reduced by ~14% in co-treatment with 20 mM GlcN and POS group (*p* < 0.05). This result demonstrates that GlcN could reduce phagocytosis at higher concentrations (20 mM). Next, we used flow cytometry to evaluate the effect of GlcN (2.5, 5.0, and 10.0 mM) treatment for 3 h and 24 h on phagocytosis in ARPE-19 cells. Consistent with these data displayed in Figure 1D, co-treatment with indicated concentrations of GlcN (2.5, 5, and 10 mM) and POS for 3 h had no significant influence on phagocytosis of ARPE-19 cells compared with the POS-treated group (Figure 1E). Furthermore, compared to the POS-treated group, there was no significant difference on phagocytosis of ARPE-19 cells after co-treatment with indicated concentrations of GlcN (2.5, 5, and 10 mM) and POS for 24 h (Figure 1F). Taken together, these results indicate that the concentrations (2.5, 5.0, and 10.0 mM) of GlcN treatment had no significant influence on the phagocytosis of ARPE-19 cells. Thus, these concentrations of GlcN were used for further experiments.

### 2.3. GlcN Induces Autophagy in ARPE-19 Cells

#### 2.3.1. Effect of GlcN on the Autophagosomes and Autophagic Markers in ARPE-19 Cells

**Figure 2:**
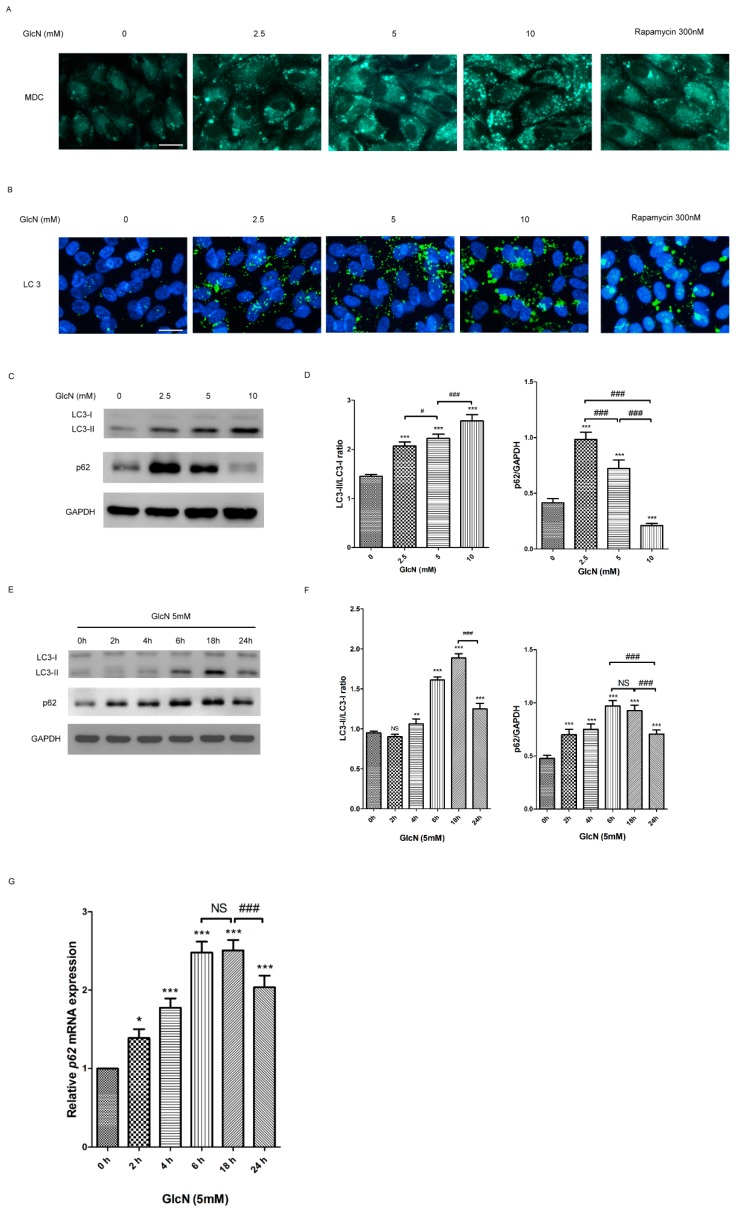
Glucosamine (GlcN) increases autophagosome and autophagic markers in ARPE-19 cells. (**A**) The number of monodansylcadaverine (MDC)-labeled vacuoles increased by either GlcN or rapamycin (Rapa) treatment in ARPE-19 cells. Cells were treated with or without 2.5 mM GlcN, 5 mM GlcN, 10 mM GlcN, or 300 nM rapamycin (Rapa) for 18 h and then stained with MDC. Rapa treatment was used as a positive control. MDC-labeled vacuoles were examined by fluorescence microscopy. Magnification, ×400; Scale bar: 20 μm. (**B**) The number of cytoplasmic LC3 puncta increased by either GlcN or Rapa treatment in ARPE-19 cells. Cells were treated with or without 2.5 mM GlcN, 5 mM GlcN, 10 mM GlcN, or 300 nM rapamycin (Rapa) for 18 h and then stained with anti-LC3 antibody. The cytoplasmic LC3 puncta were examined by fluorescence microscopy. Nuclei were counterstained with DAPI. Magnification, ×400; Scale bar: 20 μm. (**C**) Effect of GlcN on the expression of LC3-II and p62 protein in ARPE-19 cells. Cells were treated with the indicated concentrations of GlcN (0, 2.5, 5, and 10 mM) for 18 h. Whole-cell lysates were prepared and analyzed with immunoblotting using anti-LC3, anti-p62, and anti-GAPDH antibodies. (**D**) Optical density of the Western blot bands for LC3-I, LC3-II, p62, and GAPDH was analyzed. The results are represented as the mean ± SEM. The differences in the LC3-II/LC3-I ratios and p62/GAPDH in ARPE-19 cells between the groups were compared using ANOVA. Tukey’s test was used for the post hoc analysis; *** *p* < 0.001 versus the control group; ^#^ *p* < 0.05; ^###^ *p* < 0.001. (**E**) The cells were treated with 5 mM GlcN for the indicated time (0, 2, 4, 6, 18, and 24 h). Whole-cell lysates were prepared and analyzed by immunoblotting using anti-LC3, anti-p62, and anti-GAPDH antibodies. (**F**) Optical density of the Western blot bands for LC3-I, LC3-II, p62, and GAPDH was analyzed. The results are represented as the mean ± SEM. The differences in the LC3-II/LC3-I ratios and p62/GAPDH in ARPE-19 cells between the groups were compared using ANOVA. Tukey’s test was used for the post hoc analysis; ns, not significant; ** *p* < 0.01 versus the control group; *** *p* < 0.001 versus the control group; ^###^ *p* < 0.001. (**G**) The cells were treated with 5 mM GlcN for the indicated time (0, 2, 4, 6, 18, and 24 h). The levels of *p62* mRNA were quantified by using qPCR. The results are represented as the mean ± SEM. The relative *p62* mRNA expression in ARPE-19 cells between the groups were compared using ANOVA. Tukey’s test was used for the post hoc analysis; ns, not significant; * *p* < 0.05 versus the control group; *** *p* < 0.001 versus the control group; ^###^ *p* < 0.001.

Given that autophagy can be induced by GlcN in many cell types, we further investigated whether GlcN could induce the activation of autophagy in ARPE-19 cells. To determine changes in autophagy caused by GlcN treatment, we examined the effects of GlcN on the number of autophagosomes in ARPE-19 cells by monodansylcadaverine (MDC) staining. As shown in Figure 2A, there were fewer MDC-positive granules in the control group (without GlcN treatment), indicating that fewer autophagosomes existed at basal conditions. Following treatment with either GlcN or rapamycin (positive control), an increase in MDC-positive granules was observed compared to the control group, indicating that both GlcN and rapamycin increased the number of autophagosomes in cells. Next, we used Microtubule-associated protein 1 light chain 3 (LC3) immunofluorescence staining to observe the effects of GlcN on autophagosomes in cells. Consistent with MDC staining, fewer LC3-positive puncta were observed in the control group, whereas following treatment with either GlcN or rapamycin caused an increase in the number of LC3-positive puncta compared to the control group (Figure 2B). These results indicate that GlcN, like rapamycin, increased the number of autophagosomes in ARPE-19 cells. According to a previous study [36], when autophagy is induced, the cytosolic form of microtubule-associated protein 1 light chain 3-I (LC3-I) is converted into the membrane-bound form (LC3-II), which can be examined by Western blot analysis to reveal the increase of LC3-II and the LC3-II/LC3-I ratio. The p62 protein binds to both LC3 and ubiquitinated substrates, and subsequently becomes incorporated into the completed autophagosome for degradation, meaning that an increase of p62 degradation is linked to autophagy activation. As mentioned above, we used Western blot analysis to verify the effects of GlcN on the expression of LC3-I, LC3-II, and p62 proteins in cells. In Figure 2C,D, indicated concentrations (2.5, 5, and 10 mM) of GlcN treatment for 18 h increased the expression of LC3-II and the LC3-II/LC3-I ratio in a dose-dependent manner. However, the expression of p62 protein was increased in cells treated with either 2.5 or 5 mM GlcN compared to the control group and decreased in a dose-dependent manner. As shown in Figure 2E,F, GlcN treatment increased the expression of LC3-II and the LC3-II/LC3-I ratio in a time-dependent manner, with an early onset at 4 h and a peak within 18 h. The increase of p62 protein began at 2 h, reached a peak during 6 and 18 h, and then decreased at 24 h. Accordingly, 18 h was chosen as the time point for assessing the effects of GlcN for further experiments. Given that the results in Figure 2C,E show that treatment with 5 mM GlcN stimulates autophagosome formation and increases the levels of p62, we further analyzed whether GlcN treatment increased the *p62* mRNA expression by qPCR. The qPCR data show that treatment with GlcN increased the expression of *p62* mRNA from 2 h to 18 h, and then decreased it at 24 h (Figure 2G). Both Western blot (Figure 2F) and qPCR (Figure 2G) data show that GlcN increased the expression of both p62 mRNA and protein in a time-dependent manner in ARPE-19 cells. Overall, these results suggest that GlcN increases the autophagosomes and autophagic markers and has dual effect on the initial increase of the expression of p62 protein and subsequent p62 degradation in a dose-dependent manner in ARPE-19 cells.

#### 2.3.2. Effect of GlcN on Autophagic Flux in ARPE-19 Cells

**Figure 3:**
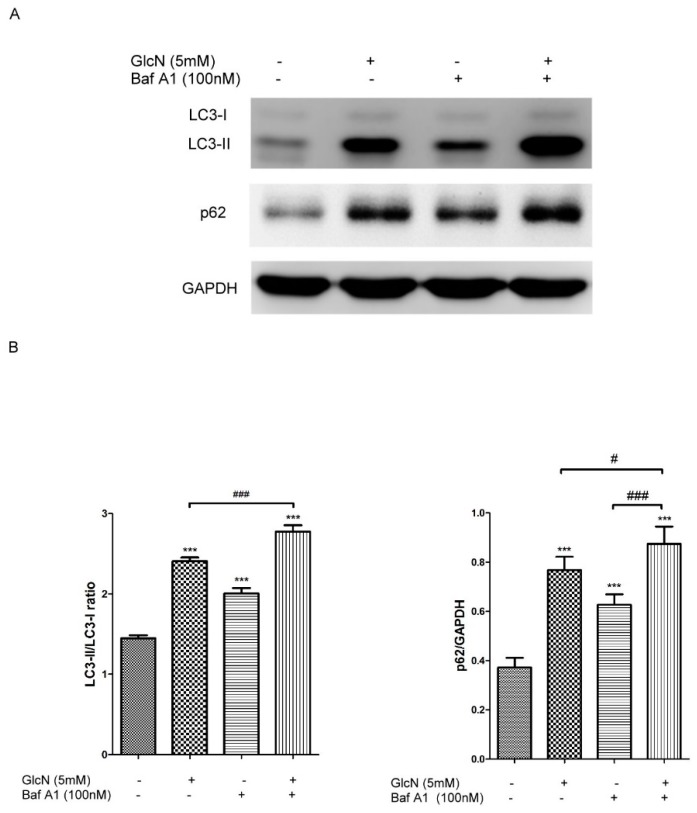
GlcN increases autophagy flux in ARPE-19 cells. (**A**) The cells were pre-treated with or without Bafilomycin A1 (Baf A1; 100 nM) for 1 h followed by co-treatment with or without GlcN (5 mM) for another 18 h. Whole-cell lysates were prepared and analyzed with immunoblotting using anti-LC3, anti-p62, and anti-GAPDH antibodies. (**B**) The optical density of Western blot bands for LC3-I, LC3-II, p62, and GAPDH was analyzed. The results are represented as the mean ± SEM. The differences in the LC3-II/LC3-I ratios and p62/GAPDH in ARPE-19 cells between the groups were compared using ANOVA. Tukey’s test was used for the post hoc analysis; *** *p* < 0.001 versus the control group; ^#^ *p* < 0.05; ^###^ *p* < 0.001.

Based on the above results (Figure 2), we further investigate whether GlcN treatment could increase autophagic flux in ARPE-19 cells. Bafilomycin A1 (Baf A1), an inhibitor of the V-ATPase, prevents maturation of autophagic vacuoles by blocking the fusion of autophagosomes with lysosomes [37]. Increased expression of LC3-II in the presence of Baf A1 is an indicator of autophagy flux [36]. To examine the effect of GlcN treatment on autophagy flux, cells were pretreated with or without 100 nM Baf A1 for 1 h and then co-treated with or without 5 mM GlcN for 18 h. As shown in Figure 3, either GlcN or Baf A1 treatment increased expression of LC3-II and the LC3-II/LC3-I ratio compared to the control group. As expected, co-treatment with GlcN and Baf A1 significantly increased the expression of LC3-II and the LC3-II/LC3-I ratio compared with either GlcN or Baf A1 treatment alone. In addition, another indicator of autophagic flux is the increase of p62 degradation [36]. Consequently, we additionally examined the expression of p62 protein in this experiment. We found that either GlcN or Baf A1 treatment increased the levels of p62 protein compared to the control group, and that co-treatment with GlcN and Baf A1 increased the levels of p62 protein higher than Baf A1 treatment alone. Consistent with above results (Figure 2F,G), this result further supports the dual effect of GlcN on the initial increase of p62 protein expression and subsequent p62 degradation in ARPE-19 cells. Taken together, these results suggest that GlcN increases autophagy flux and is able to induce autophagy in ARPE-19 cells.

### 2.4. Effect of GlcN on POS-Derived LLAF in ARPE-19 Cells

**Figure 4:**
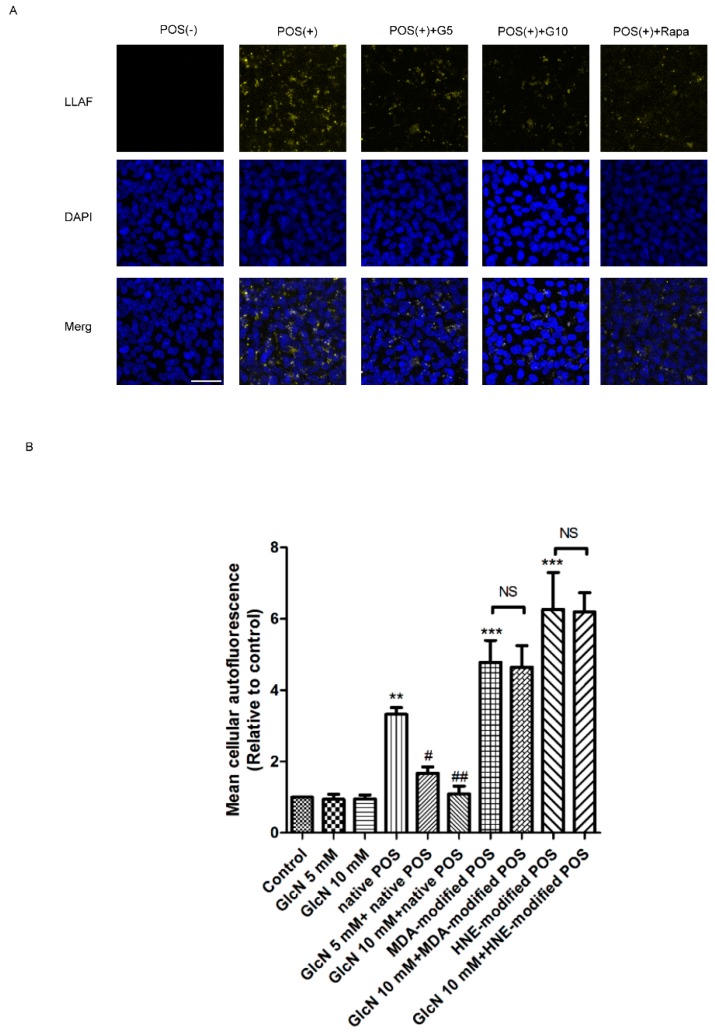
Effects of glucosamine (GlcN) on photoreceptor outer segment (POS)-derived lipofuscin-like autofluorescence (LLAF) in ARPE-19 cells. (**A**) Effect of GlcN on native POS-derived LLAF in ARPE-19 cells. Cells were examined by confocal microscopy following seven days of treatment with native POS, native POS with GlcN (5 or 10 mM), or native POS with rapamycin (Rapa; 300 nM), and were compared with untreated cells. Magnification, ×200. Scale bar: 50 μM. (**B**) Effect of GlcN on either native or modified POS-derived LLAF in ARPE-19 cells. Cells were treated with GlcN (5 or 10 mM), native POS, native POS + GlcN (5 or 10 mM), MDA-modified POS, HNE-modified POS, MDA-modified POS + GlcN (10 mM), or HNE-modified POS + GlcN (10 mM), and were compared with untreated cells. The mean fluorescence intensity (MFI) was quantified by flow cytometry following seven days of incubation. The results are represented as mean ± SEM. The differences of the MFI in ARPE-19 cells were compared between groups using ANOVA, and Tukey’s test was used for post hoc analysis; ns, not significant; ** *p* < 0.01 versus the control group; *** *p* < 0.001 versus the control group; ^#^ *p* < 0.05 versus the native POS group; ^##^ *p* < 0.01 versus the native POS group.

As shown in the above results (Figure 2 and Figure 3), we found that GlcN could induce activation of autophagy in ARPE-19 cells. It is well-known that there is a reciprocal relationship between autophagy and lipofuscin accumulation in RPE cells [19,20]. Several studies have described that the effects of autophagy induction reduce the level of LLAF, whereas autophagy inhibition leads to an increase of LLAF in POS-treated RPE cells [38,39,40,41]. These observations prompted us to further investigate the relationship between activation of autophagy and POS-derived LLAF in GlcN-treated ARPE-19 cells. First, to identity whether GlcN could influence POS-derived LLAF in ARPE-19 cells, we used confocal microscopy to observe the effects of either GlcN or rapamycin on the presence of autofluorescent granules, a characteristic feature of lipofuscin accumulation, in native POS-treated ARPE-19 cells. As shown in Figure 4A, enhanced accumulation of autofluorescent granules was seen in ARPE-19 cells treated with native POS for seven days compared to untreated control cells. Reduced autofluorescent granules were seen in cells co-treated with either GlcN or rapamycin compared to native POS-treated cells. These results indicated that treatment with either GlcN or rapamycin reduced native POS-derived LLAF in cells. According to a recent study, both lipofuscinogenesis and autophagy are interrelated processes directly associated with lysosomal function [38]. Impaired lysosomal function by Malondialdehyde (MDA)- and 4-hydroxynonenal (HNE)-modified POS could increase lipofuscinogenesis and reduce autophagy [38]. Hence, to further investigate the effect of GlcN on LLAF in either native POS or modified-POS treated ARPE-19 cells, we used flow cytometry to quantify intracellular LLAF after treatment with native, MDA-modified, or HNE-modified POS for seven days by measuring the mean fluorescence intensity (MFI). The MFI represents the quantification of intracellular LLAF. As shown in Figure 4B, the LLAF was significantly increased following treatment with native, MDA-modified, and HNE-modified POS compared to control group (*p* < 0.01, *p* < 0.001, and *p* < 0.001, respectively). Compared with the native POS-treated group, co-treatment with indicated concentrations (5 mM and 10 mM) of GlcN significantly decreased the native POS-derived LLAF in a dose-dependent manner (*p* < 0.05 and *p* < 0.01, respectively). In contrast, treatment with either MDA- or HNE-modified POS increased LLAF compared with the native POS group. Compared to either MDA- or HNE-modified POS-treated group, co-treatment with GlcN slightly decreased the LLAF, but did not reach statistically significant differences. Thus, these results show that lysosomal function disruptors MDA- or HNE-modified POS could significantly increase LLAF compared with the native POS, whereas GlcN exhibited ineffective reduction in the increase of LLAF in modified-POS treatment. Taken together, these results suggest that GlcN significantly attenuates the increase of LLAF in native POS-treated ARPE-19 cells, while impaired lysosomal function could decrease the effects of GlcN on the reduction of LLAF.

### 2.5. Relationship between GlcN-Induced Autophagy and Attenuating Effects of GlcN on Native POS-Derived LLAF in ARPE-19 Cells

#### 2.5.1. Effects of 3-MA on GlcN-Induced Autophagy

**Figure 5:**
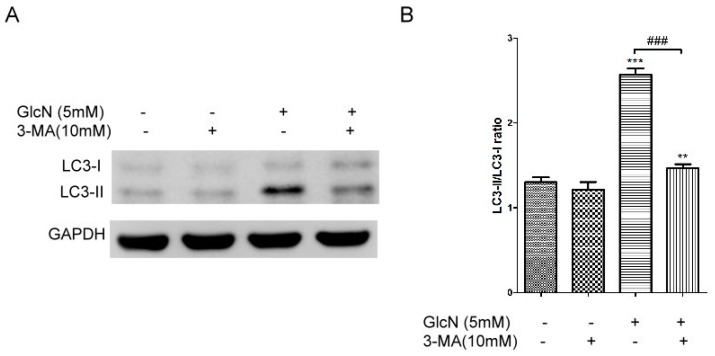
3-Methyladenine (3-MA) inhibited glucosamine (GlcN)-induced autophagy in ARPE-19 cells. (**A**) The cells were pretreated with or without 3-MA (10 mM) for 1 h followed by co-treatment with or without GlcN (5 mM) for another 18 h. Whole-cell lysates were prepared and analyzed with immunoblotting using anti-LC3 and anti-GAPDH antibodies. (**B**) Optical density of the Western blot bands for LC3-I, LC3-II, and GAPDH was analyzed. The results are represented as the mean ± SEM. The differences in the LC3-II/LC3-I ratios between groups were compared using ANOVA, and Tukey’s test was used for post hoc analysis; ns, not significant; ** *p* < 0.01 versus the control group; *** *p* < 0.001 versus the control group; ^###^ *p* < 0.001.

Given that either MDA- or HNE-modified POS could impair lysosome function to increase lipofuscinogenesis and reduce the activity of autophagy in RPE cells in vitro [38], we hypothesized that autophagy inducers could decrease the POS-derived LLAF in ARPE-19 cells. As shown in the above results, we found that treatment with GlcN effectively attenuated the increase of native POS-derived LLAF, whereas this effect could be suppressed by lysosomal function disruptors (Figure 4). As such, we asked whether the attenuating effect of GlcN on the increase of native POS-derived LLAF was related to the GlcN-induced autophagy activation. Given that previous studies have demonstrated that 3-Methyladenine (3-MA) can inhibit the GlcN-induced autophagy in vitro [33,34], we further assessed whether 3-MA could inhibit GlcN-induced autophagy in ARPE-19 cells by Western blot analysis. As shown in Figure 5, treatment with 3-MA slightly decreased the expression of LC3-II and the LC3-II/LC3-I ratio compared with the control group, though no statistically significant differences were observed. As expected, treatment with GlcN significantly increased the expression of LC3-II and the LC3-II/LC3-I ratio compared to the control group (*p* < 0.001). Compared to the group treated with GlcN alone, 3-MA significantly suppressed the increase of LC3-II expression and the LC3-II/LC3-I ratio in GlcN-treated ARPE-19 cells (*p* < 0.001). Thus, these results suggest that 3-MA inhibits the GlcN-induced autophagy in ARPE-19 cells.

#### 2.5.2. Effects of 3-MA on the Attenuating Effects of GlcN in Native POS-Derived LLAF

**Figure 6:**
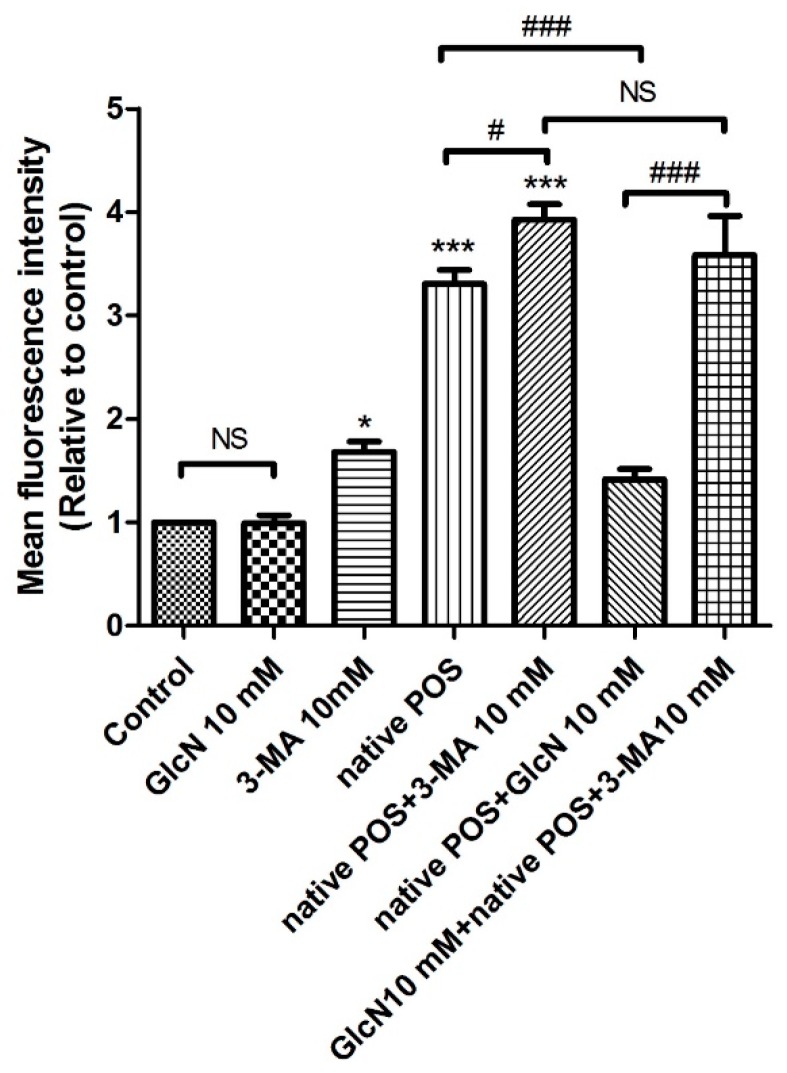
3-Methyladenine (3-MA) attenuated the effect of glucosamine (GlcN) on native photoreceptor outer segment (POS)-derived lipofuscin-like autofluorescence (LLAF) in ARPE-19 cells. Cells were treated with GlcN (10 mM), 3-MA (10 mM), native POS, native POS + 3-MA, native POS + GlcN, or GlcN + native POS + 3-MA, and were compared with untreated cells. The mean fluorescence intensity (MFI) was quantified by flow cytometry following seven days of incubation. The results are represented as the mean ± SEM. The differences of the MFI between groups were compared using ANOVA, and Tukey’s test was used for the post hoc analysis; ns, not significant; * *p* < 0.05 versus the control group; *** *p* < 0.001 versus the control group; ^#^ *p* < 0.05; ^###^ *p* < 0.001.

Based on the above results (Figure 5), we then investigated whether 3-MA could modulate the effect of GlcN on native POS-derived LLAF by flow cytometry. As shown in Figure 6, compared to the control group, LLAF was significantly increased in the 3-MA, native POS, and native POS + 3-MA group (*p* < 0.05, *p* < 0.001, and *p* < 0.001, respectively). Co-treatment with 3-MA significantly increased the native POS-derived LLAF compared with the native POS group (*p* < 0.05). Consistent with above results (Figure 5), co-treatment with GlcN significantly decreased the native POS-derived LLAF compared to the native POS group (*p* < 0.001). However, 3-MA significantly reversed the attenuating effect of GlcN on the increase of native POS-derived LLAF (*p* < 0.001). In addition, there were no statistically significant differences found between the group co-treated with native POS + 3-MA and the group co-treated with GlcN + native POS + 3-MA. Thus, these results demonstrate that 3-MA could inhibit the attenuating effect of GlcN on the increase of native POS-derived LLAF in ARPE-19 cells. Altogether, these data suggest that GlcN attenuated native POS-derived LLAF, at least in part, through inducing autophagy activation in ARPE-19 cells.

### 2.6. GlcN-Inducted Autophagy via the AMPK–mTOR Pathway

**Figure 7:**
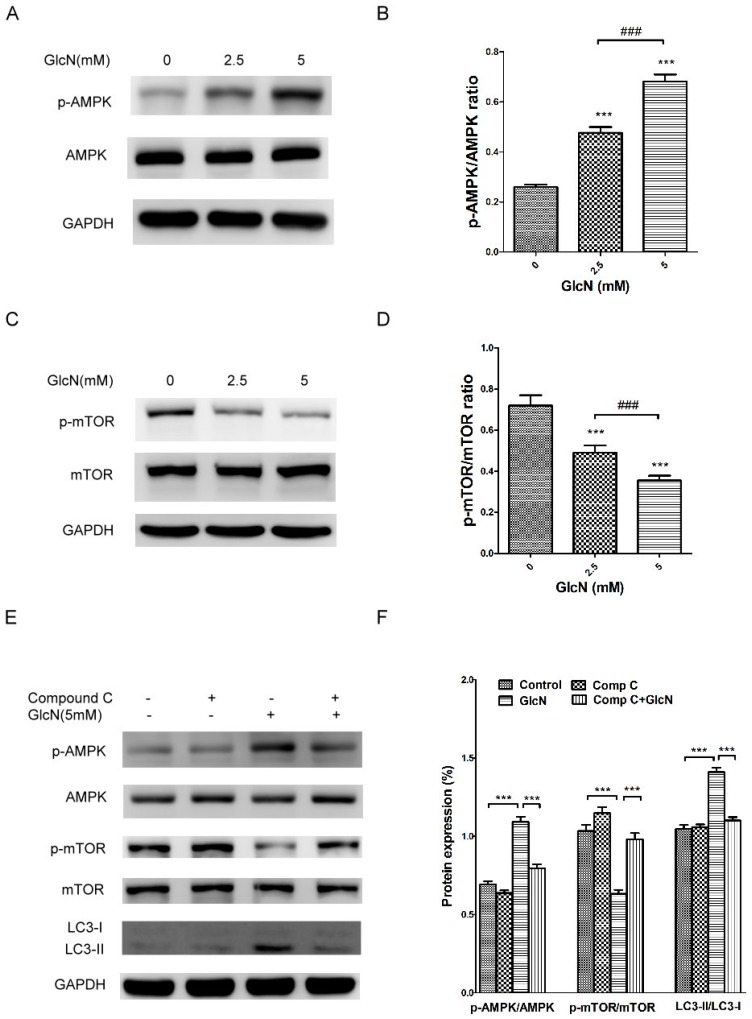
Glucosamine (GlcN)-induced autophagy via the AMPK–mTOR pathway. (**A**) GlcN decreased the level of p-mTOR protein in a dose-dependent manner in ARPE-19 cells. Cells were treated with the indicated concentrations of GlcN (0, 2.5, or 5 mM) for 18 h. Whole-cell lysates were prepared and analyzed by immunoblotting using anti-phospho-mTOR, anti-mTOR, and anti-GAPDH antibody. (**B**) The optical density of Western blot bands for p-mTOR, mTOR, and GAPDH was analyzed. The results are represented as the mean ± SEM. The differences in the p-mTOR/mTOR ratio between groups were compared using ANOVA, and Tukey’s test was used for post hoc analysis; *** *p* < 0.001 versus the control group; ^###^ *p* < 0.001. (**C**) GlcN increased the level of p-AMPK protein in a dose-dependent manner. Cells were treated with the indicated concentrations of GlcN (0, 2.5, or 5 mM) for 18 h. Whole-cell lysates were prepared and analyzed with immunoblotting using anti-phospho-AMPK, anti-AMPK, and anti-GAPDH antibodies. (**D**) The optical density of Western blot bands for p-AMPK, AMPK, and GAPDH was analyzed. The results are represented as the mean ± SEM. The differences in the p-AMPK/AMPK ratio between groups were compared using ANOVA, and Tukey’s test was used for post hoc analysis; *** *p* < 0.001 versus the control group; ^###^ *p* < 0.001. (**E**) GlcN-induced autophagy through the AMPK–mTOR pathway. Cells were pre-treated with or without compound C (Comp C; 5 µM) for 1 h followed by co-treatment with or without GlcN (5 mM) for another 18 h. Whole-cell lysates were prepared and analyzed with immunoblotting using anti-phospho-AMPK, anti-AMPK, anti-phospho-mTOR, anti-mTOR, anti-LC3, and anti-GAPDH antibodies. (**F**) The optical density of the Western blot bands for p-AMPK, AMPK, p-mTOR, mTOR, LC3-I, LC3-II, and GAPDH was analyzed. The results are represented as the mean ± SEM. The differences in the p-AMPK/AMPK, p-mTOR/mTOR, and LC3-II/LC3-I ratios between groups were compared using ANOVA, and Tukey’s test was used for post hoc analysis; *** *p* < 0.001.

Previous studies have reported that GlcN induces autophagy activation through the mTOR pathway in vitro [32,33]. The AMPK–mTOR signaling pathway plays an important role in regulating autophagy activation [42,43]. To further examine the role of AMPK–mTOR pathway in autophagy activation in GlcN-treated ARPE-19 cells, we used Western blotting analysis to detect the protein levels of p-AMPK, AMPK, p-mTOR, mTOR, LC3-I, and LC3-II. As shown in Figure 7A,B, treatment with GlcN significantly increased the p-AMPK levels in a dose-dependent manner. Because p-mTOR is downstream of AMPK, we evaluated the effect of GlcN on the levels of p-mTOR. As shown in Figure 7C,D, treatment with GlcN significantly decreased the levels of p-mTOR in a dose-dependent manner. As such, we next used compound C, an AMPK inhibitor [44], to investigate the relationship between GlcN-induced AMPK activation, mTOR suppression, and autophagy activation in cells. As shown in Figure 7E,F, compared to control cells, treatment with GlcN significantly increased p-AMPK levels, decreased p-mTOR levels, increased LC3-II levels, and increased the LC3-II/LC3-I ratio (*p* < 0.001 for each). In contrast, compared to GlcN-treated cells, compound C significantly attenuated the effect of GlcN on the increase of p-AMPK levels, decrease of p-mTOR levels, increase of LC3-II levels, and increase of the LC3-II/LC3-I ratio. This result implies that GlcN induced AMPK activation, mTOR inhibition, and autophagy activation in ARPE-19 cells, whereas compound C reversed these effects. Taken together, our results reveal that GlcN induces autophagy in ARPE-19 cells, at least in part, via the AMPK–mTOR pathway.

## 3. Discussion

**Figure 8:**
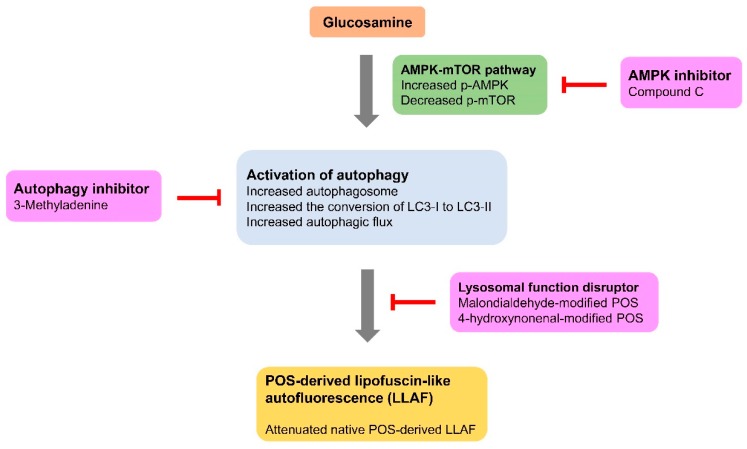
Schematic representation of the Glucosamine (GlcN)-induced autophagy on the native photoreceptor outer segment (POS)-derived lipofuscin-like autofluorescence (LLAF) and the potential pathway associated with GlcN-induced autophagy in ARPE-19 cells.

The effects of GlcN on the critical pathogenic features of the RPE in AMD and the mechanisms underlying its effects have not been investigated. In this study, we examined the effects of GlcN on autophagy induction and the levels of LLAF, both of which are important in the mechanistic basis of AMD, in ARPE-19 cells. We demonstrated that GlcN at the lower doses does not affect the phagocytosis of POS in ARPE-19 cells. We report for the first time that GlcN induces the activation of autophagy in cells and affects the increase of LLAF in POS-treated cells. We found that GlcN effectively reduces native POS-derived LLAF, but lysosomal function disruptors attenuated this effect. We additionally found that GlcN-induced autophagy plays an important role in reducing LLAF in native POS-treated ARPE-19 cells. In addition, we found that GlcN induces autophagy in ARPE-19 cells, at least in part, through the AMPK–mTOR signaling pathway (Figure 8).

Based on the results from the present study, GlcN-induced autophagy attenuates the increase of native POS-derived LLAF in ARPE-19 cells. The lysosomal function disruptors inhibited the attenuating effect of GlcN on LLAF. In addition, autophagic inhibitor suppresses the GlcN-induced autophagy and subsequently, it inhibits the attenuating effect of GlcN on LLAF. Furthermore, GlcN increases the phosphorylation of AMPK and decreases the phosphorylation of mTOR, whereas AMPK inhibitor suppresses these effects of GlcN. These results suggest that GlcN-induced autophagy is mediated, at least in part, by the AMPK–mTOR pathway.

GlcN is commonly used to treat patients with osteoarthritis. A previous study suggested that GlcN exhibits an anti-inflammatory effect in arthritis through suppressing the phagocytic activity of neutrophils [45]. GlcN (0.01–1 mM) inhibits the phagocytosis of complement-opsonized zymosan or IgG-opsonized latex particles in neutrophils [45]. As is well-known, a key function of the RPE is regular phagocytosis of shed POS to maintain the functioning of photoreceptors and to protect photoreceptors from degeneration [6]. The indigested POS is a major source of lipofuscin in RPE cells [8,9]. Thus, it is important to investigate whether GlcN affects phagocytosis of POS in RPE cells. Compared to a previous study [45], the present study showed that treatment with GlcN at lower doses (2.5, 5, and 10 mM) did not reduce phagocytosis in ARPE-19 cells in vitro. In contrast, treatment with GlcN at high doses (20 mM) reduced phagocytosis by approximately 14% in ARPE-19 cells. Therefore, this result suggests that the influence of GlcN on phagocytic activity is dependent on cell type.

Autophagy is a self-degradative process which mediates the degradation and recycling of unused and damaged intracellular components by engulfing them into autophagosomes and delivering them to the lysosome for elimination. Autophagy in RPE cells is required to maintain cellular homeostasis, and its dysregulation mediates dysfunction of RPE cells [16,17]. Moreover, it plays an important role in the clearance of accumulated deposits and the accumulation of lipofuscin in RPE cells [19,20]. Previous studies have reported that GlcN induce autophagy in various cells and animal models [31,32,33,34]. Consistent with previous reports [32,33,34], the present study shows that GlcN, like rapamycin, increased the number of autophagosome in ARPE-19 cells. GlcN treatment increased autophagy markers in ARPE-19 cells. Because the increase of autophagosomes and autophagy markers may reflect a reduction in lysosome degradation [36], the evaluation of autophagic flux is critical to resolve this issue. Our data show that GlcN increased autophagy flux, as well as induced autophagy in ARPE-19 cells. Interestingly, treatment with 10 mM GlcN increased autophagosome formation and decreased the levels of p62 protein. However, 5 mM GlcN increased autophagosome formation, but increased the levels of p62 protein. In addition, the levels of p62 protein were higher in cells co-treated with GlcN and Baf A1 compared with cells treated with Baf A1 alone. Owing to these discrepancies in the levels of p62 between Figure 2C,E and Figure 3, we assessed the expression of p62 mRNA. Our data demonstrate that GlcN increased the expression of *p62* mRNA in a time-dependent manner (Figure 2G). These data reveal that the increase of p62 protein at these GlcN concentrations (2.5 mM and 5 mM) is a result of an increase in the expression of p62 protein, not a decrease in the degradation of p62 protein. Taken together, we suggest that GlcN induces autophagy and has a dual effect on the increase of p62 expression and p62 degradation in ARPE-19 cells. In addition to mediating autophagy, p62 protein protects RPE cells from oxidative stress and is a potential therapeutic target for AMD [46]. Thus, our observation that GlcN upregulates p62 in RPE cells reveals another possible effect of GlcN that could be valuable for treating AMD. However, whether the expression of p62 protein is generally increased by GlcN treatment in primary RPE cells and other RPE cell lines requires further investigation. Furthermore, because the phagocytosis of POS induces autophagy in RPE cells [18], GlcN may enhance autophagy even further in POS-treated RPE cells. Thus, further studies examining the induction of autophagy and autophagic flux by treatment with GlcN in POS-treated cells would improve the understanding of the effect of GlcN on autophagy. Recent studies indicate that the accumulation of lipofuscin-like autofluorescent pigment occurs within native- and lipid peroxidation modified-POS treated human RPE cells in vitro [38,41], Meanwhile, treatment with autophagy inducers, such as rapamycin, could reduce early formation of LLAF in POS-treated ARPE-19 cells [41]. Consistent with these studies [38,41], our study demonstrates that treatment with native POS increased the level of intracellular LLAF in ARPE-19 cells, and that GlcN, like rapamycin, attenuated the increase of LLAF in native POS-treated ARPE-19 cells. After light exposure, lipofuscin can generate ROS and then initiate a vicious cycle, which increases lipid peroxidation and damages POS by chemical modifications, such as those by MDA and HNE [47]. MDA- and HNE-modified POS are lipid peroxidation forms in lipofuscin [47], and they are involved in lipofuscinogenesis by reducing lysosomal degradative capacity [38]. Given above study findings, we examined whether GlcN could attenuate the increase of LLAF in MDA- or HNE-modified POS-treated ARPE-19 cells. Our study demonstrates that GlcN insignificantly attenuated the increase of LLAF in MDA- and HNE-modified POS-treated ARPE-19 cells. Collectively, the present study demonstrates that GlcN attenuated the increase of native POS-derived LLAF in ARPE-19 cells, whereas MDA- and HNE-modified POS suppressed this effect. Based on a literature review [8], incomplete proteolytic digestion of POS within RPE lysosomes plays an essential role in lipofuscin accumulation, whereas RPE-lipofuscin found in AMD patients are indigestible granules and mostly composed of vitamin A-derived lipids. The indigestible/lipid-rich lipofuscin under photo-oxidative stress could lead to the formation of lipid peroxidation–modified protein in vivo, such as MDA- or HNE-modified POS. In this study, most of POS-derived LLAF came from undigested proteins and the LLAF emitted by indigestible material (MDA- or HNE-modified POS) could not be reduced by treatment with GlcN. Thus, our observation suggests that GlcN significantly induced proteolytic processes, but it could not effectively eliminate the indigestible/lipid-rich lipofuscin in the RPE of eyes with AMD. Furthermore, these results imply that GlcN might be a preventive, but not a therapeutic, agent for AMD retinas, which may consist of various lipid peroxidation products.

Furthermore, 3-MA, an autophagy inhibitor, inhibits GlcN-induced autophagy and reverses the effect of GlcN [33,34]. Accordingly, in the present study, 3-MA inhibited autophagy activation in GlcN-treated ARPE-19 cells. Recent studies have demonstrated a close interrelationship between reduced autophagy activity and increased lipofuscin accumulation in RPE cells [17,38,41]. Inhibition of autophagy by 3-MA induces lipofuscinogenesis in both RPE cells and POS-treated RPE cells [38,41]. In the present study, our observation that 3-MA increased intracellular LLAF in either ARPE-19 cells or native POS treated ARPE-19 cells is consistent with the results of previous studies [38,41]. In addition, 3-MA partially reversed the attenuating effect of GlcN on the increase in native POS-derived LLAF in ARPE-19 cells. Additionally, GlcN insignificantly attenuated the increase of LLAF in cells co-treated with 3-MA and native POS. Taken together, these results suggest that GlcN attenuates the increase in native POS-derived LLAF through autophagy activation.

Autophagy is regulated by a complex signaling network of multiple signaling pathways, in which AMPK–mTOR pathway plays a central role in the regulatory process [42,43]. AMPK is an upstream regulator of mTOR which modulates autophagy [42]. Several studies have demonstrated that GlcN can induce AMPK activation in vitro and in vivo [48,49]. In the present study, GlcN treatment increased the levels of p-AMPK protein, which is consistent with the previous findings [48,49]. In addition, recent reports have indicated that GlcN treatment can induce autophagy through the mTOR pathway in vitro [32,33]. Consistent with these studies [32,33], our data demonstrate that GlcN treatment led to a decrease in the level of p-mTOR protein in ARPE-19 cells. Additionally, compound C inhibited the effects of GlcN on the activation of AMPK, inhibition of mTOR, and activation of autophagy in ARPE-19 cells. Taken together, our results support the important role of AMPK–mTOR signaling pathway in GlcN-induced autophagy in ARPE-19 cells. Furthermore, recent studies reported that the modulation of AMPK–mTOR function is a potential target for AMD [17,41]. Further studies comparing the effects of AMPK–mTOR modulators and GlcN on phagocytosed POS and the attenuation of POS-derived LLAF in RPE cells would reveal whether the attenuating effect of GlcN on POS-derived LLAF is related to modulation of the AMPK–mTOR pathway.

ARPE-19 cells is a useful experimental platform for experiments mimicking the RPE, whereas the characteristics of RPE cells are based on various culture conditions [35,50]. The key conditions for monolayer formations of ARPE-19 cells include the plating density, ECM coating, and time of post-confluent culture [35,50]. Consistent with previous studies [35,50], the present study revealed that seven days post-confluent cultures of ARPE-19 cells mimic basic characteristics of RPE cells. In addition, Toops et al. demonstrated that polarized primary RPE monolayers possess basal autophagy activity [51]. We additionally showed that the control groups in Figure 3A,B have fewer positive MDC and LC3 puncta under this culture condition, which is consistent with a previous study [51]. As such, the ARPE-19 cell cultures behave as RPE rather than just a non-specific cell line in the present study. However, further studies are needed to replicate some of the experimental data presented here (e.g., GlcN reduces the increase of POS-derived LLAF, and GlcN induces autophagy) in well-differentiated, post-confluent primary human RPE cultures or primary RPE cultures from an animal model of AMD. The effects of GlcN on these primary RPE cells will provide us with more information on the role of GlcN on AMD.

The present study was designed to give an overview of the possible applications of GlcN to prevent the pathogenesis of AMD, such as the increase of lipofuscin in RPE cells. However, there are some limitations. For example, the phagocytosis assays are relatively routine after a 3-h time point (Figure 1D,E). FITC fluorescence after quenching indicates phagocytosis of the POS. Owing to the study design, we additionally evaluated the FITC fluorescence after treatment with POS for 24 h (Figure 1F). This time point may be long enough that the ultimate signal produced is likely a result of both phagocytosis (which increases the signal) and POS degradation (which reduces the signal). Thus, it is not certain whether the FITC signal is proportional to actual phagocytosis. As such, for long time points, another experimental method using other materials, such as fluoresceinated latex beads, might be necessary to confirm the results from FITC-labeled POS, which might resolve this concern in the present study. In addition, our results showed that 20 mM GlcN affects POS phagocytosis for 3 h, and we subsequently incubated POS daily for seven days to assess the effects of 5 mM GlcN on POS-derived LLAF in cells. Because of the long-term nature of these experiments, it is possible that some differences in GlcN-treated cells were caused by chronic impairment of POS phagocytosis, which may be undetectable in a 3-h assay. Therefore, further studies are needed to evaluate the effect of 5–10 mM GlcN on POS phagocytosis daily for seven days. Moreover, although our results suggest that treatment with either GlcN or rapamycin reduced the increase of native POS-derived LLAF in cells, it remains necessary to refine these findings. For example, examining native POS phagocytosis by performing the 3-h FITC-POS experiment after treatment with either GlcN or rapamycin for seven days would provide additional support for the effects of either GlcN or rapamycin on the decrease of POS-derived LLAF, as is presented in Figure 4A.

## 4. Materials and Methods

### 4.1. RPE Cell Culture

The human RPE cell line ARPE-19 was obtained from the American Type Culture Collection (Manassas, VA, USA). Cells were maintained in Dulbecco’s modified Eagle’s medium (DMEM)/F-12 (Invitrogen-Gibco, Grand Island, NY, USA) supplemented with 10% fetal bovine serum (FBS; Invitrogen-Gibco) and antibiotic–antimycotics (Invitrogen-Gibco) at 37 °C in 5% CO_2_. Cells were cultured according to a previously described procedure [50]. Briefly, cells were seeded at 1.66 × 10^5^/cm^2^ in Matrigel-coated wells (wells coated with Geltrex^®^ LDEV-free reduced growth factor basement membrane matrix), according to the manufacturer’s protocols (Thermo Fisher Scientific, Waltham, MA, USA) and incubated for 1–7 days. The characteristics of RPE cells were investigated by Western blot analysis and immunocytochemistry (Figure 1). The culture medium was replaced twice weekly. All experiments were performed in complete medium unless otherwise stated.

### 4.2. RPE Cell Treatment

For every experiment, 1.66 × 10^5^/cm^2^ RPE cells were seeded onto Matrigel-coated wells and cultured for an additional seven days unless otherwise specified. Monolayers of RPE cells were preincubated for 1 h with or without 300 nM rapamycin (Sigma-Aldrich, St. Louis, MO, USA) as an autophagy inducer [43], 100 nM bafilomycin A1 (Baf A1; Sigma-Aldrich) as a vacuolar type H(+)-ATPase (V-ATPase) inhibitor [37], 10 mM 3-methyladenine (3-MA; Sigma-Aldrich) as an autophagy inhibitor [52], or 5 μM compound C (Sigma-Aldrich) as an AMPK inhibitor [44]. Cells were further incubated with or without GlcN (Sigma-Aldrich) at the respective concentrations (2.5, 5.0, 10.0, or 20 mM) for the indicated period at 37 °C. The added compounds were retained during the entire period of the experiment.

### 4.3. Western Blot Analysis

Western blotting was used to detect ZO-1 (Zonula occludens-1, a tight junction protein) [53], RPE65 (Retinal pigment epithelium-specific protein 65 kDa, an RPE differentiation marker) [53], MerTK (Mer tyrosine kinase, a phagocytic receptor) [53], LC3-I/II (microtubule-associated protein 1 light chain 3-I/II, an autophagy marker) [36], p62 (sequestosome 1, an autophagy marker) [36], p-mTOR and mTOR (mammalian target of rapamycin, mTOR pathway markers) [43], p-AMPK and AMPK (AMP-activated protein kinase, AMPK pathway markers) [43], and anti-glyceraldehyde 3-phosphate dehydrogenase (GAPDH, an internal control) in the cell lysates. Cells were washed twice with phosphate-buffered saline (PBS) and detached by scraping. Cells were pelleted at 1000× *g*, resuspended, and sonicated in cold lysis buffer (50 mM Tris-HCl [pH 7.5] 2% sodium dodecyl sulfate (SDS), and 1 mM phenylmethylsulfonyl fluoride). Insoluble materials were removed by centrifugation at 12,000× *g* for 15 min at 4 °C. Protein concentration was determined using the bicinchoninic acid method (BCA; Pierce, Rockford, IL, USA). Bovine serum albumin (BSA) was used as a protein concentration standard. One-dimensional Sodium dodecyl sulfate–polyacrylamide gel electrophoresis (SDS-PAGE) was used to resolve 20 µg of cell lysates. Separated proteins were transferred onto polyvinylidene difluoride (PVDF) membranes (Immobilon; Millipore, Bedford, MA, USA) blocked with 5% (*w*/*v*) milk for 1 h at room temperature. The membranes were further incubated overnight at 4 °C with the respective antibodies diluted 1:1000 unless otherwise stated in tris-buffered saline containing Tween-20 (TBST, 0.1% at 1×). The antibodies used in the present study were anti-ZO-1 (Thermo Fisher Scientific), anti-RPE65 (Abcam, Cambridge, MA, USA), anti-MerTK (Abcam), anti-LC3 (Cell Signaling Technology, Danvers, MA, USA), anti-p62 (Cell Signaling Technology), anti-phospho-AMPK (Thr172, Abcam), anti-AMPK (Abcam), anti-phospho-mTOR (Ser2448, Cell Signaling Technology), anti-mTOR (Cell Signaling Technology), and GAPDH (1:5000; Santa Cruz Biotechnology, Santa Cruz, CA, USA). These antibodies (LC-3, p62, p-AMPK, and p-mTOR) were used to investigate the relevance of the autophagy and AMPK–mTOR pathways. After incubation, membranes were washed and incubated with a horseradish-peroxidase-conjugated secondary antibody (1:25,000; Jackson ImmunoResearch Laboratories, West Grove, PA, USA) for 1 h at room temperature. Proteins were visualized using enhanced chemiluminescence (ECL) procedure (ECL reagent; Millipore, Billerica, MA, USA). Western blot images were acquired with a UVP BioSpectrum 500 and analyzed by VisionWorks LS software (UVP, Upland, CA, USA).

### 4.4. Immunocytochemistry

ARPE-19 cells cultured on Matrigel-coated coverslips and then incubated in the presence or absence of GlcN. Cells were washed, fixed in 4% paraformaldehyde, treated with 0.1% Triton X-100 for 10 min at room temperature, and incubated with 5% BSA in PBS for 1 h at room temperature. Primary antibodies used were anti-ZO-1 (1:100), anti-RPE65 (1:100), and anti-LC3 (1:100). Alexa Fluor^®^ 488 donkey anti-rabbit immunoglobulin G (IgG) and Alexa Fluor^®^ 594 goat anti-mouse IgG antibodies (1:200; BioLegend, San Diego, CA, USA) were used as secondary antibodies. Nuclei were counterstained with 4′,6′-diamidino-2-phenylindole (DAPI, Sigma-Aldrich). Preparations were mounted in 70% glycerol. The protein expressions of ZO-1, RPE65, and LC3 were examined using a fluorescence microscope (CKX41; Olympus Corporation, Tokyo, Japan). The LC3 immunofluorescence staining was used to observe the number of LC3-positive puncta, which corresponds to the number of autophagosomes [36].

### 4.5. Monodansylcadaverine Staining

Monodansylcadaverine (MDC) staining is an autophagosome indicator, as has been described previously [54]. Briefly, ARPE-19 cells were cultured on Matrigel-coated coverslips for seven days. Following incubation with or without 2.5 mM, 5 mM, or 10 mM GlcN, or 300 nM rapamycin for 18 h, the cells were incubated with 0.05 mM MDC (Sigma-Aldrich) in PBS at 37 °C for 10 min. After incubation, cells were washed four times with PBS and images were captured using a fluorescence microscope (CKX41; Olympus Corporation) equipped with an ultraviolet filter (excitation filter: 330–385 nm, barrier filter: 420 nm).

### 4.6. RNA Isolation and Quantitative Polymerase Chain Reaction (qPCR)

Total RNA was extracted from ARPE-19 cells according to the manufacturer’s protocol (TRIzol Reagent; Invitrogen-Gibco). For cDNA synthesis, total RNA (2 μg) was reverse transcribed with a High-Capacity cDNA Reverse Transcription Kit (Applied Biosystems, Foster City, CA, USA), according to the manufacturer’s instructions. Subsequently, quantitative polymerase chain reaction (qPCR) for p62 was performed according to the protocol of the TaqMan assay. Briefly, the qPCR was performed in triplicate, using 8-tube strips with 1 μL of TaqMan probe, 10 μL of master mix (TaqMan Universal Master Mix II, Applied Biosystems), 4 μL of cDNA (100 ng), and 5 μL of diethylpyrocarbonate-treated water (Invitrogen, Carlsbad, CA, USA). The thermal cycling profile consisted of an initial temperature of 50 °C for 2 min, followed by a denaturation step at 95 °C for 10 min, and then 40 successive cycles at 95 °C for 15 s and 60 °C for 1 min. The primer set of the TaqMan assay for SQSTM1/p62 was Hs01061917_g1 (Applied Biosystems). The amount of target mRNA relative to the expression of the endogenous control gene and relative to values from the control group was calculated using the relative quantitation method. mRNA expression levels were normalized using the expression of *GAPDH* (Hs02758991_g1, Applied Biosystems) as the endogenous housekeeping gene.

### 4.7. POS Isolation

Porcine eyes were purchased from a local slaughterhouse and transported on ice. POSs were isolated according to a previously described procedure [38,55]. Briefly, the anterior half of the eye was dissected and the vitreous and retina were removed. Isolated retinas were agitated in KCl buffer (0.3 M KCl, 10 mM HEPES, 0.5 mM CaCl_2_, 1 mM MgCl_2_) with 48% *w*/*v* sucrose at pH 7.0 and centrifuged at 7000 rpm for 5 min. The supernatant containing the POS was filtered through sterile gauze, diluted 1:1 with KCl buffer without sucrose, and centrifuged at 5000 rpm for 7 min. The pelleted POSs were repeatedly washed in PBS and stored at −80 °C until further use.

### 4.8. Malondialdehyde and 4-Hydroxynonenal Modification of POS

Malondialdehyde (MDA) and 4-hydroxynonenal (HNE) were prepared by acid hydrolysis from 1,1,3,3-tetramethoxypropane (Sigma-Aldrich) and (E)-4-hydroxynonenal-dimethylacetal (Sigma–Aldrich), respectively, as described in studies by Kaemmerer et al. and Krohne et al. [38,56]. MDA- and HNE-modified POS serve as lysosome function disruptors [38,56]. In brief, POSs were incubated with 20 mM MDA or 5 mM HNE overnight at room temperature on a shaker. Unbound MDA and HNE were removed by washing repeatedly with PBS. Concentration of modified proteins resulting from this procedure was measured by a method described previously [38,56]. Protein content of POS preparations was measured using Bradford protein assay. The modified POSs were stored at −80 °C until further use.

### 4.9. Phagocytosis Assay

Phagocytosis was measured according to a previously established method [57,58]. Briefly, pelleted POSs were suspended in culture media and diluted to a concentration of 1 × 10^9^ POS/mL. Before analysis, POSs were labeled with fluorescein-5-isothiocyanate (FITC “Isomer I”; Thermo Fisher Scientific) according to the manufacturer’s protocol. Cells were cultured on Matrigel-coated 96-well plates for seven days. After preincubation with or without GlcN (2.5, 5, 10, or 20 mM,) for 24 h, RPE cells were incubated with FITC-POS (10 POS per cell) for 3 h under culture conditions and then the FITC fluorescence was quenched with 0.4% Trypan blue in PBS for 10 min at room temperature. Cells were rinsed four times with PBS containing 1 mM MgCl_2_ and 0.2 mM CaCl_2_. The total fluorescence was measured using a Synergy H4 hybrid multi-mode microplate reader (BioTek, Winooski, VT, USA) at an excitation wavelength of 485 ± 20 nm and an emission wavelength of 528 ± 20 nm.

### 4.10. Flow Cytometry-Based Phagocytosis Assay

Modified flow cytometry-based phagocytosis assay was performed according to a previously described method [59,60]. Briefly, cells were seeded on Matrigel-coated dishes and preincubated with or without GlcN (2.5, 5, or 10 mM) for 24 h. Cells were incubated with FITC-POS (4 µg/cm^2^ POS) for 3 h and 24 h at 37 °C. Cells were rinsed with 1× Dulbecco’s PBS (with calcium; Invitrogen) and treated with trypsin for 8 min to detach cells and release the bound POS. DMEM/F12 with 2% FBS (Invitrogen-Gibco) was added to neutralize the trypsin. Samples were diluted 1:1 with Fluorescent Activated Cell Sorting (FACS) staining buffer containing anti-DRAQ5 (1:2500; Cell Signaling) and analyzed immediately on an FACSCalibur flow cytometer (BD Biosciences, San Jose, CA, USA). Cells were gated based on DRAQ5 labeling and 10,000 events were recorded.

### 4.11. Induction of POS-Derived LLAF in Vitro

POS-derived LLAF was induced based on a published method [38]. Briefly, cells were seeded at a confluent density of 1.66 × 10^5^/cm^2^ and cultured for seven days. For induction of LLAF, cells were incubated with 4 µg/cm^2^ POS daily for seven days. Culture medium and incubated POSs were renewed daily.

### 4.12. Observation of LLAF in Vitro by Confocal Microscopy

For confocal microscopy imaging, cells were seeded on Matrigel-coated coverslips, cultured for seven days, and incubated with POS for seven days. Where indicated, 5 mM GlcN, 10 mM GlcN, or 300 nM rapamycin were co-incubated with or without POS. Culture medium, incubated POS, GlcN, and rapamycin were renewed daily for seven days. After incubation, cells were repeatedly washed to remove non-internalized POS and fixed with 4% paraformaldehyde. Nuclei were counterstained with DAPI (Sigma-Aldrich). Preparations were mounted in 70% glycerol and images were captured using a confocal microscope with an excitation filter wavelength ranging from 467 to 498 nm and an emission filter wavelength ranging from 513 to 556 nm (LSM880, Carl Zeiss MicroImaging, Inc., Thornwood, NY, USA).

### 4.13. Quantification of LLAF by Flow Cytometry

LLAF was measured by flow cytometry as described previously [38]. In brief, cells were seeded on Matrigel-coated dishes, cultured for seven days, and were then incubated with POS, MDA-modified POS, or HNE-modified POS for seven days. Where indicated, 5 mM GlcN, 10 mM GlcN, or 10 mM 3-MA were co-incubated with or without POS, MDA-modified POS, or HNE-modified POS. Culture medium, incubated POS, MDA-modified POS, HNE-modified POS, GlcN, and 3-MA were renewed daily for seven days. Cells were washed with PBS three times, detached by treating with trypsin for 1 min, and analyzed on a FACSCalibur flow cytometer (BD Biosciences) using the FITC channel (excitation laser wavelength, 488 nm; detection filter wavelength, 530/30 nm). For each sample run, 10,000-gated cells were recorded and analyzed. The mean fluorescence intensity was reported as the mean of LLAF for each sample.

### 4.14. Statistical Analysis

Significant differences between two groups were analyzed by a paired *t* test. Normally distributed continuous variables were compared with a one-way ANOVA. When a significant difference between the groups was apparent, multiple comparisons of their means were made with Tukey’s procedure. Data are presented as means ± SEM. Each result is representative of at least three independent experiments. All statistical assessments were evaluated at the 0.05 level of significance. Statistical analyses were performed with GraphPad Prism version 5.01 for Windows (GraphPad Software, La Jolla, CA, USA).

## 5. Conclusions

In conclusion, the present study provides evidence to suggest that GlcN does not affect phagocytosis of POS, but induces autophagy in ARPE-19 cells. GlcN effectively decreased LLAF in native POS-treated cells, but not in MDA- or HNE-modified POS-treated cells. The reduction in LLAF was associated with GlcN-induced autophagic activation. GlcN induced autophagy, at least in part, through the AMPK–mTOR pathway. Collectively, our findings suggest that GlcN administration is a potential preventive option for lipofuscin-related retinal degeneration, such as that found in AMD.

## Abbreviations

AMD	Age-related macular degeneration
RPE cells	Retinal pigment epithelial cells
POS	Photoreceptor outer segment
GlcN	Glucosamine
LLAF	Lipofuscin-like autofluorescence
GA	Geographic atrophy
ROS	Reactive oxygen species
ZO-1	Zonula occludens-1
RPE65	Retinal pigment epithelium-specific protein 65 kDa
MerTK	Mer tyrosine kinase
GAPDH	Glyceraldehyde 3-phosphate dehydrogenase
MDC	Monodansylcadaverine
LC3	Microtubule-associated protein 1 light chain 3
p62	sequestosome 1
Rapa	Rapamycin
Baf A1	Bafilomycin A1
MDA	Malondialdehyde
HNE	4-hydroxynonenal
3-MA	3-Methyladenine
AMPK	AMP-activated protein kinase
mTOR	mammalian target of rapamycin
Comp C	Compound C
